# Electroencephalographic Signature of Negative Self Perceptions in Medical Students

**DOI:** 10.7759/cureus.22675

**Published:** 2022-02-28

**Authors:** Richard M Millis, Justin Arcaro, Allison Palacios, Grace L Millis

**Affiliations:** 1 Department of Pathophysiology, American University of Antigua, St. John's, ATG; 2 Department of Psychology, Loyola University Maryland, Baltimore, USA

**Keywords:** mood regulation, depression prevention, psychosocial interactions, quantitative electroencephalography, frontal alpha asymmetry

## Abstract

Frontal alpha asymmetry (fAA) is purported to be a neurophysiological marker for anxiety and depression. Higher left frontal alpha EEG voltage is associated with lower left and higher right frontal cerebral cortical activation, indicative of right-sided fAA. This pilot study tests the hypothesis that greater left-sided frontal alpha voltage is associated with negative thoughts about oneself. A group of eight healthy 28-41-year-old right-handed male medical students were subjected to an extensive interactive self-report inventory (ISI) evaluating perceptions of their psychosocial interactions. Quantitative EEG (qEEG) was performed with eyes closed. Computations of fAA and related parameters were based on measurements in the alpha bandwidth (8-13 Hz) at the left frontal F7 and right frontal F8 scalp electrodes. fAA was the percent difference between mean voltages at F8 minus that at F7. Significance of associations between fAA and the ISI scores was determined by Pearson’s product-moment correlation coefficient, at P≤0.05. “Depressed” scores were positively correlated with right-sided fAA (P=0.01). “Relaxed” (P=0.05), “regulated” (P=0.02), “cooperative” (P=0.05) and “dependent scores” (P=0.004) were negatively correlated with right-sided fAA. These findings imply that right-sided fAA may be associated with more perceptions of “depressed” psychosocial interactions involving negative thoughts about oneself, as well as, more reliance on others (“dependence” score), less sharing (“cooperative” ISI score), less trust (“regulated” ISI score) and less initiative (“relaxed” ISI score). These results support the hypothesis that right-sided fAA may identify individuals with a predilection for negative thoughts about themselves and other negatively-valenced perceptions of their psychosocial interactions.

## Introduction

Neural oscillations in the alpha range (8-12 Hz) can be observed at any site of the scalp overlying the brain, and are, generally, inversely related to the activation level of the underlying cerebral cortex [1]. A low alpha wave voltage is observed when eyes are open and cortical regions are actively processing information [2]. In contrast, high alpha wave voltage is associated with the resting state with eyes closed, reflecting the activity of the brain’s default mode network [3]. Frontal alpha asymmetry (fAA) refers to the average difference in alpha-bandwidth voltage between the right and left areas of the scalp overlying the frontal lobes [4]. Decades of research reveal associations between fAA and affective processing of information and certain psychopathologies. The earliest demonstration of fAA shows a relationship between greater activation of the left than the right cerebral hemispheres when observing emotion-laden televised scenes rated as positive [5]. In unprecedented fashion, this suggests the dominance of the left hemisphere for positive emotions and the dominance of the right hemisphere for negative emotions. In terms of alpha wave voltage, greater activation of the left frontal lobe is associated with lower frontal alpha voltage on the left than on the right, and is interpreted as dominant hemispheric, left-sided fAA [6,7]. Conversely, greater activation of the right frontal lobe results in lower alpha frontal voltages on the right than on the left, interpreted as non-dominant hemispheric, right-sided fAA. In contrast to positive ratings of emotion-laden televised scenes, negative ratings of such scenes are usually associated with right-sided fAA [8]. It is customary to compute fAA as the right-sided voltage minus the left-sided voltages; hence, right-sided fAA is commonly reported as a negative value whereas left-sided fAA is a positive one. The specificity of fAA is suggested by findings that alpha wave voltages measured at scalp electrodes overlying the right and left parietal and occipital lobes fail to discriminate between positive and negative ratings of emotion-laden televised scenes [8].

When changes in affect involve depressed mood, they often reflect the “approach-avoidance” paradigm for analyzing human personalities and behavior [9]. This widely-applied behavioral paradigm, originated in the 1930s by Kurt Lewin, one of the founders of modern social psychology, is based on the human tendency to assign value to outcomes specifically distinguishing between people who “approach” desired outcomes and are associated with positive affective states versus those who “avoid” negative outcomes and are associated with negative affective states [10]. High-tech, 21st-century computer-based applications of the approach-avoidance paradigm are currently in use. For example, using a touchscreen for selection of approach vs. avoidance behavioral options to evaluate body image issues, study subjects exhibited a significant bias for selection of “approach” directed toward overweight bodies and “avoidance” of underweight bodies, correlated with their subclinical eating disorder symptoms [11]. “Approach” personality traits are often shown to reflect left-sided alpha asymmetry [9]. In left-sided alpha asymmetry, the left frontal alpha voltages are smaller than the right, indicating relatively more activation in the left, dominant frontal lobe than in the right, non-dominant frontal lobe. Conversely, avoidance traits are reported to reflect right-sided alpha asymmetry. In right-sided alpha asymmetry, the right frontal alpha voltages are smaller than the left, indicating relatively more activation in the right frontal, non-dominant hemisphere. In that regard, more right-sided fAA has been found in cohorts of depressed individuals than in non-depressed controls [12].

Depression is a complication of numerous neuropsychiatric, psychosocial and medical conditions, including drug abuse, aggression, violence, suicide, and intergenerational mental illness [13]. Current antidepressant and psychosocial therapies are, largely, ineffective as long-term preventive medicine strategies for depression [14]. Screening for depressed mood could, therefore, be advantageous for preventing the sequelae of untreated depression and improving human performance, especially for persons engaged in high-stress occupations or high-stakes activities [15]. Because right-sided fAA is purported to be a neurophysiological marker for anxiety and depression, screening for fAA might be useful for identifying individuals at risk for emotional maladjustment. Academic underachievement in medical school is a high-stakes endeavor that is known to unmask predilections for negative-valenced emotions. The present investigation is, therefore, designed as a pilot study to determine whether fAA is correlated with negative thoughts about oneself in a non-randomized, self-selected group of male medical students.

## Materials and methods

This study was approved by the American University of Antigua College of Medicine Institutional Review Board (IRB) of the American University of Antigua Research Council (approval #: 011217). Informed consent was obtained from the study participants. Of ten first-semester male medical students who were recruited, eight completed the study. All the subjects self-reported right-handedness. They were subjected to 10 min of quantitative EEG (qEEG) measurements at 19 standard electrode sites during eyes-open (EO) and eyes-closed (EC) conditions (Brain Master Discovery 20, Brain Master Technologies, Inc., Bedford, OH, USA). The EO data was acquired while the subjects were staring at a black dot on a blank white wall. All qEEG data were acquired at the same time of day, 8-10 AM, after overnight fasting, with subjects seated upright in a darkened room. The subjects were instructed to refrain from drugs such as alcohol, marijuana, and caffeine use for 24 hours prior. According to self-report, none of the subjects were taking prescription medicines or recreational drugs within the prior month. Preliminary findings that EO conditions, known to inhibit the magnitude of alpha brain wave voltage in healthy humans, resulted in fAA changes that were not uniformly reproducible. qEEG measures were, therefore, evaluated for the EC condition only. The mean ± standard deviations (SD) of the amplitudes, expressed in µV, were measured for the following standard qEEG frequencies: delta (2-4 Hz), theta (4-7 Hz), alpha (8-12 Hz), and beta (13-30 Hz) after manual editing using the New Mind Maps online editing tool (New Mind Technologies, Roswell, GA, USA). For this study, the mean alpha voltages recorded at electrode locations F7 (left) and F8 (right) were used to compute frontal alpha and beta asymmetry (fAA) according to the following formula: [(F8-F7)/F8) x 100]. The rationale for using F7 and F8 for measuring fAA was that these qEEG recording sites overly the inferior frontal gyrus, an important site of “mirror neurons” in humans, thought to be important in processing neural information relevant to psychosocial interactions [16]. Negative asymmetry values were indicative of right-sided, non-dominant hemispheric alpha asymmetry resulting from greater activation of the right frontal cortex. Negative alpha asymmetry was defined as qEEG recordings wherein the mean alpha voltage at the left frontal F7 scalp electrode was larger than that at the symmetrical right frontal F8 scalp electrode. Positive asymmetry values were indicative of left-sided, dominant hemispheric alpha asymmetry. Positive alpha asymmetry was defined as qEEG recordings wherein the mean alpha voltage at the right frontal F8 scalp electrode was larger than that at the symmetrical left frontal F7 scalp electrode. F8-F7 alpha coherence, F8-F7 alpha-phase-locking and F8, F7 dominant alpha (mode) frequency were computed electronically by the Brain Master Discovery 20 software. 

Within eight hours after the qEEG measurements, each of the study subjects completed an Interactive Self-Report Inventory (ISI, New Mind Technologies, Roswell GA, USA) online at the New MInd Maps website (https://www.newmindmaps.com). The subjects and research assistants were blinded to the main purpose of the study; to correlate their ISI responses with their qEEG measurements. They were told that their qEEG measurements and their ISI responses were being correlated with their first-semester examination scores. The ISI is a questionnaire requiring responses about the subject’s psychosocial interactions. Validity of the ISI is based on Pearson’s product-moment and Spearman’s rank correlation coefficients computed from the scaled scoring of answers to the ISI by male and female subjects (N=2,721) ranging from 16 to 92 years of age. This reference population was drawn from all socioeconomic groups and ethnicities undergoing neurobiofeedback training in approximately 300 clinics in all geographical areas of the USA. It is considered that this population is as close to being a random sample as possible without engaging in formal targeting procedures using stratified sampling methods. 

Data from the reference population were collected anonymously from a total of 136 ISI items based on two meta-dimensions of approach and avoidance behaviors plus 14 sub-dimensions of psychosocial interactions, totaling 16 dimensions of measurement using 5-point Likert scales. The ISI dimensions are each composed of 5-16 questions on average with anxiety and depression measures containing 15 and 16 items, respectively. Anxiety and depression scales were based on average positive correlations of 86% (r=0.86) with the Beck Depression Inventory (BDI) when comparisons were run for cross-validation. These scales were included in the measures to validate approach and avoidance validity and to enhance cross-validation with other psychometrics. Discriminant validity of meta-scaling was confirmed by correlating factor loadings of approach and avoidance with depression and anxiety. Pearson’s and Spearman’s correlations between responses to the ISI items indicative of avoidant vs. interactive (approach) behavioral temperament and each of the 14 ISI dimensions of psychosocial interaction are presented in Table 1 (New Mind Technologies, Roswell GA, USA).

**Table 1:** Correlation coefficients for interactive self-report inventory (ISI) dimensions of psychosocial interactions. Pearson = Pearson’s product-moment correlation coefficient; Spearman = Spearman’s rank correlation coefficient. Reference population N=2,721, df = 2,719; *P<0.05, **P<0.01, ***P<0.001.

Behavioral Paradigm	Approach	Approach	Avoidant	Avoidant
ISI Dimension	Pearson	Spearman	Pearson	Spearman
Anxious	-0.259***	-0.265***	0.420***	0.409***
Depressed	-0.303***	-0.301***	0.453***	0.440***
Relaxed	0.342***	0.334***	-0.264***	-0.267***
Inhibited	-0.339***	-0.336***	0.578***	0.570***
Regulated	0.103***	0.094***	-0.063**	-0.065**
Impulsive	-0.050*	-0.053**	0.234***	0.239***
Passive	-0.101***	-0.105***	0.226***	0.222***
Assertive	0.400***	0.387***	-0.325***	-0.318***
Flexible	0.286***	0.288***	-0.205***	-0.233
Perfectionistic	-0.159***	-0.154***	0.381***	0.377***
Cooperative	0.375***	0.367***	-0.229***	-0.256***
Competitive	0.009	0.016	0.179***	0.190***
Independent	0.133***	0.188***	-0.059***	-0.072***
Dependent	0.033	0.024	0.248***	0.244***

Table 2 summarizes the rationales for each of the 16 ISI dimensions queried (New Mind Technologies, Roswell GA, USA). 

**Table 2:** Interactive self-report inventory (ISI) dimensions of psychosocial interactions.

ISI Dimension	Rationale
Anxious	Individuals will have fearful thoughts (r>0.8, Beck’s Anxiety Inventory)
Depressed	Individuals will have negative thoughts about themselves (r>0.8, Beck’s Depression Inventory
Relaxed	Individuals will invite interaction
Inhibited	Individuals will not self-disclose and not engage others to build relationships
Regulated	Individuals will build trust
Impulsive	Individuals will violate norms and erode trust in others and consequently themselves
Passive	Individuals go along with others all of the time and will violate themselves by not getting the resources they need
Assertive	Individuals will act to secure resources
Flexible	Individuals can adjust to change and adapt to circumstance to overcome adversity and challenge
Perfectionistic	Individuals will frustrate themselves and others in attempting to get things done to secure resources
Cooperative	Individuals will encourage others to participate and improve outcomes through sharing resources
Competitive	Individuals will discourage others from participating and diminish their self-esteem
Independent	Individuals demonstrate confidence, feel self-empowered and actively define clear boundaries
Dependent	Individuals will not take initiative and will not generate conflict by attempting to have others secure their resources for themselves
Interactive (Approach)	High scores on assertion, cooperation, independence, relaxed, and regulated are considered positive dimensions associated with social approach. In terms of established psychosocial concepts, these dimensions are related to positive outcomes, high self-esteem, social accuracy and acquisition of social resources.
Avoidant	High scores on inhibition, passivity, perfectionism, competitiveness, and dependence are considered negative dimensions associated with social retreat. In terms of established psychosocial concepts, these dimensions are related to negative outcomes and low self-esteem.

Statistical analysis

Significance of correlations between fAA and each of the ISI dimensions of psychosocial interactions was determined by computing the Pearson product-moment correlation coefficient, at P≤0.05. Specificity of measurements and findings related to F8-F7 fAA, alpha coherence, alpha phase-locking, and alpha dominant (mode) frequency was evaluated by comparing them to the same measurements at qEEG recording sites surrounding frontal F7 and F8, at the orbitofrontal, prefrontal F3 and F4 and at the temporal T5 and T6 electrodes.

## Results

Seven of the eight study subjects exhibited negative F8-F7 mean voltages and one subject had positive F8-F7 mean voltage ranging from -124.3 µv to +1.5 µv, (mean -43.5 ± 44.4 µv) indicating a predominance of higher left-sided, dominant hemispheric frontal alpha voltage, lower left frontal cortical activation and, therefore, a predominance of right-sided fAA. Table 3 lists the correlation coefficients for the relationships between fAA and ISI scores for 16 dimensions of the subjects’ perceptions of their psychosocial interactions. 

**Table 3:** Correlations between interactive self-report inventory (ISI) scores and F8-F7 frontal alpha asymmetry. r = Pearson’s correlation coefficient. *Significant at P≤ 0.05; **significant at P≤ 0.01.

ISI Dimensions	Correlation (r)	P-value
Anxious	-0.55	0.16
Depressed	-0.83	0.01**
Relaxed	0.69	0.05*
Inhibited	-0.37	0.37
Regulated	0.79	0.02*
Impulsive	-0.48	0.23
Passive	-0.28	0.50
Assertive	0.33	0.42
Flexible	0.44	0.27
Perfectionistic	0.02	0.96
Cooperative	0.69	0.05*
Competitive	-0.02	0.96
Independent	0.21	0.69
Dependent	0.88	0.004**
Interactive	0.62	0.10
Avoidant	-0.26	0.53

**Figure 1:**
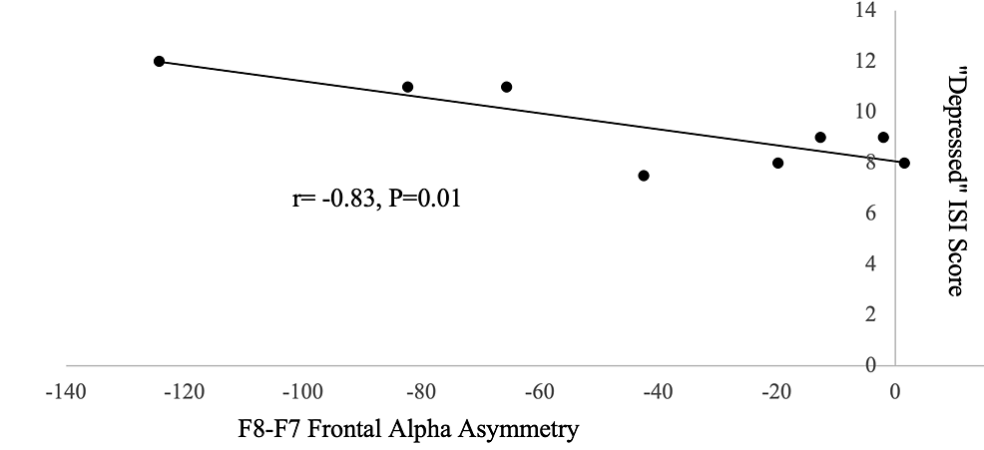
Association of “depressed” ISI score with frontal alpha asymmetry. Correlation between quantitative EEG measurement of frontal alpha asymmetry and interactive self-report inventory (ISI) scoring of perceptions of psychosocial interactions as “depressed” in 8 male medical students. Negative frontal alpha asymmetry values indicate larger mean alpha voltage recorded from F7 on the scalp overlying the left frontal cerebral cortex than from F8 overlying the right cortex, interpreted as lower left than right frontal alpha activity; therefore, right-sided frontal alpha asymmetry.

Figure 1 demonstrates the association between self-reported “depressed” ISI scores and the computation of fAA.

“Depressed” ISI scores were negatively correlated with asymmetric F8-F7 alpha voltages (r= -0.83, P=0.009, Figure 1), “Depressed” ISI scores were also negatively correlated with F8-F7 alpha coherence (r= -0.75, P=0.03 and with F8-F7 alpha phase-locking (r= -0.88, P=0.006). “Depressed” ISI scores were positively correlated with F8 alpha dominant mode frequency (r=0.87, P=0.006). Mean alpha voltages and mode alpha frequencies at F7 were not significantly correlated with “depressed” ISI scores (P>0.1).

**Figure 2:**
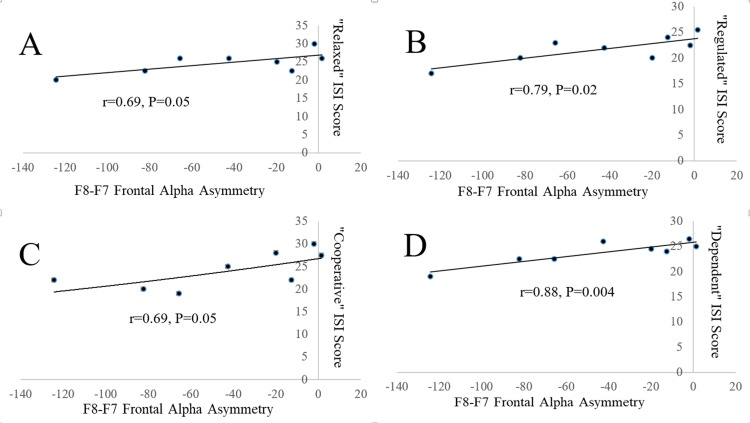
Figure 2. Associations of ISI scores with frontal alpha asymmetry. Correlations between quantitative EEG measurement of frontal alpha asymmetry and interactive self-report inventory (ISI) scores of perceptions of psychosocial interactions as “relaxed” (Panel A), “regulated” (Panel B), “cooperative” (Panel C) and “dependent” (Panel D) in 8 male medical students. Negative frontal alpha asymmetry values indicate larger mean alpha voltage recorded from F7 on the scalp overlying the left frontal cerebral cortex than from F8 overlying the right cortex, interpreted as lower left than right frontal alpha activity; therefore, right-sided frontal alpha asymmetry.

Figure 2 shows that “relaxed” (r=0.69, P=0.05), “regulated” (r=0.79, P=0.02), “cooperative” (r=0.69, P=0.05) and “dependent” (r=0.88, P=0.004) ISI scores were positively correlated with asymmetric F8-F7 voltages. “Regulated” and “dependent” ISI scores were also positively correlated with F8-F7 alpha phase-locking (r=0.72, P=0.04 and r=0.86, P=0.006, respectively). 

## Discussion

Models of fAA

The “approach-interaction vs. avoidance-withdrawal” model of fAA proposes that neural systems mediate motivational trait tendencies to approach or withdraw from novel emotional stimuli [17]. In terms of fAA, the qEEG recording site with greater alpha voltage is shown to exhibit less cortical activation than the hemisphere with less alpha voltage [1-4]; hence, the relationship between frontal alpha voltage and cortical activation is inverse. Relatively greater right frontal alpha voltage and less right frontal cortical activation (i.e., left-sided fAA) is associated with approach-related emotions (e.g. love, attraction, enthusiasm, joy); whereas, greater left frontal alpha voltage and less left frontal cortical activation (i.e., right-sided fAA) is associated with withdrawal-related emotions (e.g. fear, sadness, anxiety) [18,19]. This model overlaps substantially with the valence model, which assumes that the attractiveness or aversiveness of an emotional response valence of emotions is more important than motivational tendencies associated with the emotion [20,21]. Relatively greater left frontal activation (left-sided fAA) is, therefore, associated with positively-valenced emotions, and relatively greater right frontal activation (right-sided fAA) is associated with negatively-valenced emotion [22,23]. Positive emotions are linked to approach-related motivation and negative emotions are related to withdrawal-related motivation, with a few notable exceptions. In a study of anger, a negative valence is shown to be associated with an approach tendency; anger is favored by alpha activity in the left rather than the right frontal cortex [24]. Hence, this anomaly cannot be explained by the valence model. In sum, these findings suggest that fAA reflects the direction of the motivation rather than the valence of the emotion. In the present study, seven of the eight subjects exhibited right-sided fAA, tendencies for more negative thoughts about oneself (“depressed” scores) and more reliance on others (“dependent” scores), as well as, less sharing (“cooperative” scores), trust (“regulated” scores) and initiative (“relaxed” scores) on an extensive ISI questionnaire about the subjects’ perceptions of their own psychosocial interactions. These perceptions were all significantly correlated with more F8-F7 right-sided fAA. It is noteworthy that half of the subjects, four of eight, exhibited either a small amount of left- or right-sided fAA, less than 20%.

Trait versus state

fAA has been studied as a trait and state measure associated with personality, emotion, and some psychopathology. As a trait measure, fAA represents characteristics of the individuals possessing them, and that certain asymmetries are possessed by individuals as relatively stable traits [25]. These traits appear to modulate state measures such as emotional reactivity and are predicted to serve as diatheses that can increase the risk for psychopathology [26,27]. Some studies have examined state-dependent changes in fAA, with fluctuations in asymmetries that coincide with fluctuations in affective states. These may be thought of as changes in the EEG that are responsive to specific environmental conditions, elicited by viewing different emotional expressions, for example. One study has shown increased left frontal activation in response to a sincere and involuntary smile (i.e. Duchenne smile) relative to smiles that are voluntary and considered insincere (i.e. Pan-Am smile) [28,29]. Addressing the issue of stability pertaining to trait-like qualities, fAA shows a high internal consistency (Cronbach’s alpha ranging from .81 to .90) and good test-retest stability (intra-class correlations ranging from .44 to .71 across three weeks of measurements) [30]. From a developmental perspective, there appears to be a high correlation between alpha asymmetry recorded in infants at different stages of their development and in young children at high risk for depression [31]. Across four measurement periods, Hagemann et al. have shown that more than half of the variance in asymmetry measures could be due to individual differences of a stable trait [32,33]. In the present study, “depressed” ISI scores were significantly correlated with F8-F7 fAA, a qEEG measure of cortical specialization, F8-F7 alpha coherence and phase-locking, measures of cortical interconnectivity, and F8-F7 alpha dominant frequency, a measure of cortical efficiency. A similar pattern was found for some of the other ISI dimensions with significant correlations between the subjects’ perceptions and several frontal alpha qEEG parameters. These findings appear, therefore, to support the hypothesis that the fAA findings reported herein may prove to be a robust reflection of the subjects’ personality traits. 

Personality and social behavior

fAA has predicted internalizing and externalizing difficulties in children and crying behavior in response to maternal separation. Infants who cry in response to maternal separation seem to exhibit greater right frontal activation (right-sided fAA) at rest than those who do not cry during such separation [34]. In adults, fAA is typically related to global positive and negative affect in response to films or slides with greater right frontal activation (right-sided fAA) associated with more intense negative affect and greater left frontal activation (left-sided fAA) with more intense positive affect [35]. 

The trait and withdrawal theories of fAA are likely to have consequences for traits associated with personality and social behavior. For example, studies in adults show that individuals with lower measures on sociability display relatively greater right frontal activation and therefore right-sided fAA [36]. This fits well with research showing greater right frontal activation (right-sided fAA) in children who score lower on measures of social competency and children with higher scores on social competency display greater left frontal activation and therefore left-sided fAA [37]. Schmidt (1999) found that asymmetry is related to measures of shyness in children and is positively associated with greater right frontal activation and therefore right-sided fAA [38]. Introversion-extroversion is a commonly studied personality dimension due to its linkage to cortical arousal. Higher cerebral blood flows are shown among introverts; thereby, supporting the arousal hypothesis that assumes more cortical arousal in introverts than in extroverts [39,40]. One study demonstrates differences between groups of introverts and extroverts; the extroverts being three times more likely to have larger frontal alpha voltages [41], indicative of lower cortical arousal. In the present study, the association of right-sided fAA, indicative of lower right than left frontal alpha voltage and higher right than left frontal arousal suggests the hypothesis that, in addition to negative thoughts about oneself and other negatively-valenced perceptions of psychosocial interactions, personality extroversion vs. introversion may also be associated with F8-F7 fAA. 

Neuroticism

Another commonly studied dimension of personality is neuroticism. Neuroticism increases one’s sensitivity to fear and distress [42,43]. Individuals with high neuroticism scores are prone to emotional responses to stress that fosters avoidant behaviors including anxiety, anger, and panic [44]. This makes neuroticism a major personality risk factor for the development of psychopathology. High levels of neuroticism are associated with lower thresholds for reporting negative emotions [45] and positive correlations with negative affect [46]. Healthy individuals exhibiting decreased cortical activity and increased alpha voltage in the right hemisphere (left-sided fAA) are shown to have higher neuroticism scores, thereby suggesting a significant association between neuroticism and fAA [27]. Neuroticism correlates with the valences of fear, disgust, sadness, and surprise, but not with happiness, anger, and neutral faces [47]. More importantly, there appears to be a positive correlation with mid-frontal asymmetry and neuroticism, and an interaction between neuroticism and increased right-frontal activity (right-sided fAA) that can affect the valence of fear [27]. These findings suggest that neuroticism and mid-frontal fAA may be risk factors for certain pathologies. It is noteworthy that seven of the present study subjects exhibited varying amounts of right-sided fAA which could have also been a marker for neuroticism, perhaps an additional, potentially useful marker for self-reported “depressed” and other negatively-valenced perceptions of psychosocial interactions in medical students and others exposed to similar environmental stressors. 

Psychopathology

fAA may predict risk for psychopathology [47] by virtue of its ability to discriminate between patients with depression and control subjects [48]. Further evidence of this can be found in studies where less left frontal asymmetry (i.e., either no asymmetry or relatively more right-sided fAA) discriminates infants whose mothers are diagnosed with major depression [49]. These studies corroborate earlier findings using the BDI where higher scores on the BDI are shown to be associated with relatively greater right frontal activation and therefore right-sided fAA [50]; with similar findings among clinically diagnosed patients [51]. The majority of findings in patients with depression reveal some underlying stability across time, with some variation across occasions of assessment that cannot be fully explained. It is possible that trait asymmetry may serve as a diathesis for emotion-related psychopathology, suggesting a process by which an individual’s affective style may put them at risk for emotional imbalances and/or depression-related disorders. 

Significance

Against the backdrop of the COVID-19 pandemic, the physician shortage crisis becomes more pronounced. Current models project a total physician shortage of between 54,100 and 139,000 physicians by 2033, which may be underestimated due to the short and long-term impact of COVID-19; consequences include educational pipeline issues, workforce exits, and specialty demand shifts [reference]. Further, demand for physicians in underserved populations could rise by an additional 74,100 to 145,000 physicians should they face fewer access barriers [52]. As such, the role of foreign medical graduates, who are uniquely equipped to serve the needs of underserved populations, to supplement the physician workforce becomes paramount. Interventions to identify depression and anxiety in medical students by means of fAA and to appropriately target associated negative perception of psychosocial interactions in medical students, specifically foreign medical students, is not exclusively a moral imperative, but rather in the best interest of public health. 

Feelings of depression and anxiety are high amongst all medical students. However, when considering the specific challenges faced by foreign medical students namely, the stigma of being trained at a foreign medical institution and the difficulty of matching a U.S. residency program, it follows that inherent negative perceptions of their psychosocial interactions may be exacerbated. The research on prevalence of anxiety and depression in foreign medical students is limited at best. For this reason, this research identifying predilections for emotional imbalance, depression, and related sequelae through qEEG screening provides a unique opportunity to provide a targeted approach to improve student performance on a micro level, and to additionally contribute to the quality of the medical field at large through its focus on foreign medical students. 

Significant correlations between right-sided fAA and negative thoughts about oneself, reliance on others, as well as less sharing, trust, and initiative, both justifies identifying at-risk students and introducing pilot programs that target these negative-valenced emotions due to their high prevalence among medical students. Early identification of students with predilections for anxiety and depression may be the target of stress management and resilience training as early as their first year. Stress management and resilience trainings, dubbed “SMART” programs, have been introduced in various hospital settings to reduce stress and improve quality of life. However, the opportunity to efficaciously integrate such programs in medical school curriculums as well as other high-stress graduate degree programs (e.g. law school) remains.

Many medical educational institutions are equipped with educational enhancement and psychological counseling services to support those students who are identified as having predilections for anxiety and depression. Apart from interventions that seek to provide tools for managing negative feelings, there is an opportunity to improve the way students think similar to methods employed by cognitive behavioral therapy. However, to change one’s thinking patterns relies on recognition of problematic thinking as was identified in this study through qEEG and survey methods.

Burnout, anxiety, depression, and impostor syndrome, wherein students doubt their academic success and maintain fears of being exposed as a fraud, have all received much attention in recent years as factors that negatively impact academic outcomes and quality of life. Using qEEG screening for fAA couple with traditional survey methods to identify psychosocial at-risk students provides a novel justification for and reform of medical education.

Limitations of the study

The main limitation of the present investigation is that it is designed as a pilot study utilizing a relatively small N (number of human subjects). The relatively small N predisposes the findings to type II statistical error. Such statistical error is unavoidable in pilot studies, especially those involving human subjects, usually because of funding limitations. Nevertheless, pilot studies like the present one are invaluable, and often necessary, to provide the framework for “pushing the envelope” and developing novel hypotheses to be tested in larger studies with larger “N” to overcome the type II error, inherent in pilot studies. The limitation due to the small N is, however, overcome by the findings of very high correlation coefficients (r=0.69-0.88). This level of correlation suggests the potential for significant associations between fAA and five of the sixteen self-reported ISI measures of psychosocial interactions. Based on these correlation coefficients, squaring the correlation coefficients yields relatively high R2 values (R2= 0.48-0.77). Remembering that correlation does not imply causation, the R2 values x 100 provides an estimate of the percentage of variations in self-reported psychosocial interactions explained, although not caused, by the observed variations in fAA. In the present study, a substantial proportion, 48% to 77%, of the variation in self-reported psychosocial interactions are explained by the fAA variations. Because of these limitations, the findings reported herein should be interpreted cautiously pending confirmation by a larger study.

## Conclusions

The present pilot study tests the hypothesis that right-sided fAA is associated with negative thoughts about oneself in a cohort of first-semester male medical students. The results of this study demonstrate the potential for finding self-reported negative thoughts about oneself, as well as other negatively-valenced perceptions of a person’s psychosocial interactions in conjunction with right-sided fAA, a previously-described EEG signature for anxiety and depression. Greater left-sided, dominant hemispheric frontal alpha voltages indicative of lesser left frontal activation and, therefore, right-sided fAA were found to be associated with more negative thoughts about oneself (“depressed” scores) and more reliance on others (“dependence” scores), as well as, less sharing (“cooperative” scores), trust (“regulated” scores) and initiative (“relaxed” scores) on an extensive interactive self-inventory questionnaire. The subjects were studied during their adjustment to high-stakes academic and psychosocial challenges upon matriculation to medical school in a foreign country. The finding of self-reported “depressed” perceptions of the subjects’ psychosocial interactions positively correlated with right-sided fAA. This finding suggests that qEEG screening for fAA coupled with a questionnaire probing a person’s perceptions of their psychosocial interactions may be able to identify predilections for emotional imbalance, depression, and related sequelae in medical students and, perhaps, other populations exposed to similar environmental stressors.
